# Mortality Prediction Using SaO_2_/FiO_2_ Ratio Based on eICU Database Analysis

**DOI:** 10.1155/2021/6672603

**Published:** 2021-11-08

**Authors:** Sharad Patel, Gurkeerat Singh, Samson Zarbiv, Kia Ghiassi, Jean-Sebastien Rachoin

**Affiliations:** ^1^Cooper University Hospital, Camden, NJ, USA; ^2^Piedmont Columbus Regional, Columbus, GA, USA; ^3^University of Missouri, St. Louis, MO, USA

## Abstract

**Purpose:**

PaO_2_ to FiO_2_ ratio (P/F) is used to assess the degree of hypoxemia adjusted for oxygen requirements. The Berlin definition of Acute Respiratory Distress Syndrome (ARDS) includes P/F as a diagnostic criterion. P/F is invasive and cost-prohibitive for resource-limited settings. SaO_2_/FiO_2_ (S/F) ratio has the advantages of being easy to calculate, noninvasive, continuous, cost-effective, and reliable, as well as lower infection exposure potential for staff, and avoids iatrogenic anemia. Previous work suggests that the SaO_2_/FiO_2_ ratio (S/F) correlates with P/F and can be used as a surrogate in ARDS. Quantitative correlation between S/F and P/F has been verified, but the data for the relative predictive ability for ICU mortality remains in question. We hypothesize that S/F is noninferior to P/F as a predictive feature for ICU mortality. Using a machine-learning approach, we hope to demonstrate the relative mortality predictive capacities of S/F and P/F.

**Methods:**

We extracted data from the eICU Collaborative Research Database. The features age, gender, SaO_2_, PaO_2_, FIO_2_, admission diagnosis, Apache IV, mechanical ventilation (MV), and ICU mortality were extracted. Mortality was the dependent variable for our prediction models. Exploratory data analysis was performed in *Python*. Missing data was imputed with Sklearn Iterative Imputer. Random assignment of all the encounters, 80% to the training (*n* = 26690) and 20% to testing (*n* = 6741), was stratified by positive and negative classes to ensure a balanced distribution. We scaled the data using the Sklearn Standard Scaler. Categorical values were encoded using Target Encoding. We used a gradient boosting decision tree algorithm variant called XGBoost as our model. Model hyperparameters were tuned using the Sklearn RandomizedSearchCV with tenfold cross-validation. We used AUC as our metric for model performance. Feature importance was assessed using SHAP, ELI5 (permutation importance), and a built-in XGBoost feature importance method. We constructed partial dependence plots to illustrate the relationship between mortality probability and S/F values.

**Results:**

The XGBoost hyperparameter optimized model had an AUC score of .85 on the test set. The hyperparameters selected to train the final models were as follows: colsample_bytree of 0.8, gamma of 1, max_depth of 3, subsample of 1, min_child_weight of 10, and scale_pos_weight of 3. The SHAP, ELI5, and XGBoost feature importance analysis demonstrates that the S/F ratio ranks as the strongest predictor for mortality amongst the physiologic variables. The partial dependence plots illustrate that mortality rises significantly above S/F values of 200.

**Conclusion:**

S/F was a stronger predictor of mortality than P/F based upon feature importance evaluation of our data. Our study is hypothesis-generating and a prospective evaluation is warranted. *Take-Home Points*. S/F ratio is a noninvasive continuous method of measuring hypoxemia as compared to P/F ratio. Our study shows that the S/F ratio is a better predictor of mortality than the more widely used P/F ratio to monitor and manage hypoxemia.

## 1. Introduction

Management of hypoxia is an integral part of the intensive care unit (ICU) care. Patients in the ICU present with a wide variety of pathologies requiring varying degrees of oxygenation support. Evaluation and management of hypoxia are achieved through various forms of monitoring, including partial pressure of oxygen (PaO_2_) from an arterial blood gas analysis and pulse oximetry for oxygen saturation (SaO_2_).

The Berlin definition for Acute Respiratory Distress Syndrome (ARDS) includes the PaO_2_/FiO_2_ (P/F) ratio as a diagnostic criterion [1]. Most cutoffs for ARDS interventions are based on the P/F ratio [2,3]. Measuring PaO_2_ requires an arterial blood gas (ABG) analysis, an invasive and potentially cost-prohibitive clinical setting procedure with limited resources [4]. ABG measurement overuse has been recognized as a problem for 20 years now, leading to practice guidelines to curb this testing [5]. PaO_2_ values can vary significantly from one blood gas draw to another, and given the relative infrequency of checks, this can lead to erroneous conclusions and interventions [6,7]. Furthermore, considering the current COVID-19 pandemic, frequent blood gas checks may increase the risk of infection transmission. Many of these dogma-based processes in the ICU warrant a renewed risk-and-benefit analysis in the postpandemic scenario.

SaO_2_ is a continuously available parameter, which correlates well with PaO_2_. PaO_2_ alone is nebulous and must be considered in the context of the degree of oxygenation support. P/F ratio provides information about the pulmonary gas exchange adjusted for the quantity of oxygen delivered. SaO_2_/FiO_2_ (S/F) ratio can be calculated easily and can be considered a noninvasive alternative to P/F. A strong correlation between S/F and P/F has been reported in the available literature. Brown et al. found that PaO_2_/FiO_2_ ratios could accurately be imputed with SaO_2_/FiO_2_ (S/F) ratios through nonlinear equations, with clinical equivalence, which can be verified by comparing mortality [8]. S/F correlates with P/F for diagnosing ARDS in medical and surgical patients [9–11].

Although the S/F ratio has good accuracy and is continuously available, the S/F ratio is not a standard assessment tool for hypoxia in the ICU. There have been attempts to utilize S/F when resources are limited, where ABG may not be readily available [10]. The current evidence suggests that the S/F ratio correlates well with P/F and is comparable in ARDS diagnostic performance. S/F ratio has the advantage of being easy to calculate, noninvasive, continuous, cost-effective, and reliable, with potentially low risk of exposure, and avoids iatrogenic anemia [11]. Despite the aforementioned obvious benefits of using S/F over P/F, the data assessing clinical outcomes exists but remains sparse [9–12]. A diagnostic test's utility can partially be measured by the ability to discriminate accurately between outcomes of interest; as intensivists, the outcome of interest is often mortality. Using a machine-learning approach, we aim to retrospectively estimate the relative predictive capacity of S/F and P/F in measuring ICU mortality. Our assessment is novel. We hope to demonstrate the predictive capacity of S/F in a heterogeneous ICU population, including surgical, medical, mechanically ventilated, and nonmechanically ventilated patients. Our purpose is to demonstrate the noninferiority for mortality predictive ability of S/F relative to P/F with the ambition to promote practice change towards a less resource-laden method to assess hypoxia.

## 2. Methods

### 2.1. Ethics Statement

This study analyzed a publicly available, anonymized database with preexisting institutional review board (IRB) approval.

### 2.2. Sample Selection

**Figure 1:**
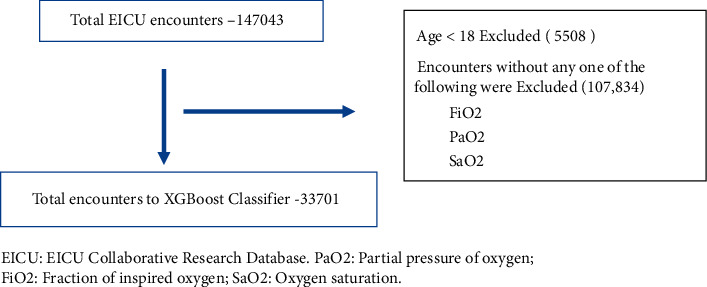
Inclusion flowchart. EICU: EICU Collaborative Research Database. PaO2: partial pressure of oxygen; FiO2: fraction of inspired oxygen; SaO2: oxygen saturation.

The eICU Collaborative Research Database is a multicenter intensive care unit database with data from over 200,000 ICU admissions monitored by eICU programs [13]. The eICU database comprises 200,859 patient unit encounters for 139,367 unique patients admitted between 2014 and 2015 from 208 hospitals located throughout the US. From the eICU encounters, stays involving adult patients (18 years and above) were included (Figure 1). Patients at all levels of oxygen and mechanical support were included. The ranges of oxygen requirements include nasal cannula to mechanical ventilation and ECMO. Patients with no admission day PaO_2_ or FiO_2_ were excluded. The variables age, gender, SaO_2_, PaO_2_, FIO_2_, admission diagnosis, Apache IV, mechanical ventilation (MV), and ICU mortality were extracted from the database [14]. PaO_2_ and FiO_2_ were drawn from the worst arterial blood gas (ABG) on day 1 of admission. SaO_2_ was measured every minute, but the final recorded value was the five-minute median value. We used the first SaO_2_ measurement recorded for the admission. Mortality was the dependent variable for our prediction models. Using SaO_2_, PaO_2_, and FiO_2_, we created two new features using the ratios SaO_2_/FiO_2_ (S/F) and PaO_2_/FiO_2_ (P/F). SaO_2_, PaO_2_, FiO_2_, S/F, gender, admission diagnosis, mechanical ventilation (Vent), and P/F were the final features used during the algorithm's training and testing. Our feature importance rankings are the physiologic parameters, including SaO_2_, PaO_2_, FiO_2_, P/F, and S/F.

### 2.3. Experimental Methods

**Figure 2:**
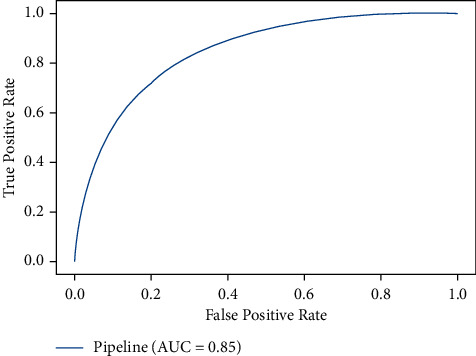
Final AUC for the test set of the hyperparameter optimized model. Pipeline: Iterative Imputer, Label Encoding, Target Encoding, Standard Scaler, and XGBoost Classifier.

The missing data were imputed with Sklearn Iterative Imputer (Version 0.23.2) [15]. Random assignment of all the encounters, 80% to the training (*n* = 26690) and 20% to testing (*n* = 6741), was stratified by positive and negative classes to ensure a balanced distribution.  Figure 2 shows the class balance for the primary outcome. The data were scaled using the Sklearn Standard Scaler. Admission diagnosis was encoded via the library Category_encoder with the Target Encoding method [16]. All predictive models depicted in this paper were instances of the XGBoost gradient boosted tree model, implemented in *Python* [17]. XGBoost is a tree ensemble method that builds progressively on the loss generated by weak decision tree base learners. A baseline XGBoost model was trained, followed by training of the final model with optimized hyperparameters. Model hyperparameters were tuned using the Sklearn RandomizedSearchCV with tenfold cross-validation. The hyperparameters chosen to optimize were colsample_bytree, gamma, max_depth, subsample, min_child_weight, and scale_pos_weight.

The XGBoost predictive models were trained and tested using repeated/stratified *K* cross-validation (*K* = 10). In this validation paradigm, the data were partitioned into ten random folds, and outcomes were distributed in equal proportions in each fold to reduce bias. Each of the ten models trained was then tested on the hold-out test set partitioned before hyperparameter tuning. The final metrics reported were averages of the five models.

Our dataset is imbalanced. An imbalanced dataset has a large difference between the majority and minority outcome classes. In our cohort, the number of patients who survived was larger than that of those who expired. Imbalanced datasets are common in medical databases and can negatively affect machine-learning classification performance. The Area Under the Receiving Operator Curve (AUC) was used as a goodness-of-fit test for our model's predictive performance. AUC was chosen as our primary metric as it is known to be relatively agnostic to minority and majority class occurrence differences [18]. Additionally, Accuracy, Recall, and Precision were reported.

Once the final model was trained, feature selection using three techniques, SHAP, Eli5, and the built-in feature importance within XGBoost, was performed [19–22]. Our primary means of assessing feature importance in this study is via the SHAP library, which derives importance using Shapley values. Shapley values are based on the idea that the outcome of each possible combination of features should be considered to determine the significance of a single feature. The Eli5 library derives feature importance via permutation importance. Values are shuffled within the dataset for each feature, predictions are generated by the model, and score change is calculated. The prediction score change of each feature is then ranked, and feature importance is derived. The XGBoost model provides feature importance ranking, which uses gain as the default method for calculation. Gain implies the relative contribution of the corresponding feature to the model calculated by taking each feature's contribution for each tree in the model. A higher value of this metric than the other features implies that it is more important for generating a prediction. Partial dependence plots were created to illustrate S/F values and the relative probability of death. This plot gives the curve representing how much the variable affects the final prediction at which variable value.

## 3. Results

Feature distribution stratified by mortality is demonstrated in Table 1. The feature value ranges and distributions were complex and at times multimodal, which are best displayed via violin plots. Violin plots provide both the interquartile range distributions and the probability distributions, the latter of which cannot be ascertained by the traditional box plot. Overall feature distribution is displayed via Violin plots (Supplemental Figures 1–6). Additionally, patients were stratified as mechanically ventilated (*n* = 27382) and nonmechanically ventilated (*n* = 5873), as displayed in Table 1. Admission diagnosis distribution is displayed in a descending order based upon the frequency in Supplemental Figure 6, with missing values demonstrated in Supplemental Figures 7 and 8.

Using the training data, we performed fivefold cross-validation on every combination of the hyperparameter values. There were 405 different hyperparameter combinations, and, with 10-fold cross-validation, a total of 2020 models were fit on the training data. The evaluation metric used to determine the best performing hyperparameter combination was AUC. The hyperparameters selected to train the final models were as follows: colsample_bytree of 0.8, gamma of 1, max_depth of 3, subsample of 1, min_child_weight of 10, and scale_pos_weight of 3.

The XGBoost base model had a final AUC of 0.84 and 0.84 on the training set and test set, respectively. The hyperparameter optimized model had AUC scores of 0.84 and 0.85 on the training set and test set, respectively. Given the minimal to no difference between the training and test scores, it is reasonable to assume that our model did not overfit. Performance metrics of the final model are shown in Table 2 and Figure 2. Base model scores are available in Supplemental Figure 9.

**Figure 3:**
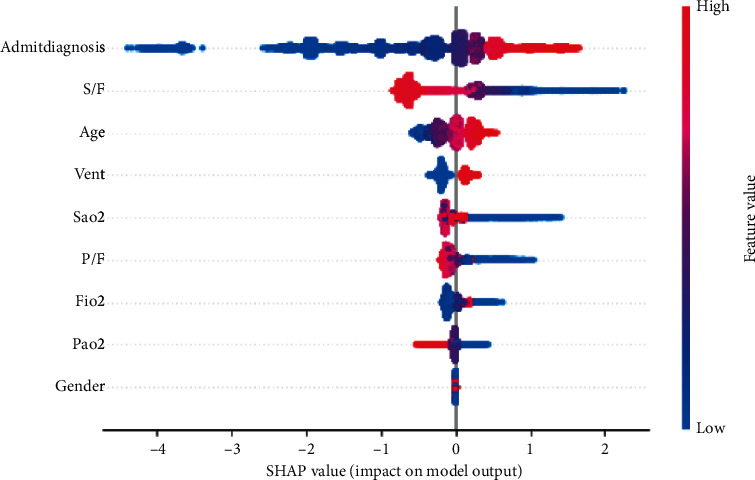
SHAP table demonstrating feature importance ranked in descending order on *y*-axis. *x*-axis with the SHAP values. Negative SHAP value predicts a low probability of death at a high or low value of that feature value (blue = low value; red = high value). Positive SHAP value demonstrates a high probability of mortality at that feature value. Low S/F values (blue) predict a high likelihood of death, whereas high values (red) predict a low likelihood. PaO_2_: partial pressure of oxygen; FiO_2_: fraction of inspired oxygen; SaO_2_: oxygen saturation; P/F: PaO_2_ and FiO_2_ ratio; S/F: SaO_2_ and FiO_2_ ratio; S/F: SaO_2_/FiO_2_, P/F: PaO_2_/FiO_2_; Vent: mechanical ventilation.

**Figure 4:**
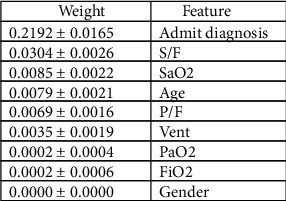
Feature importance ranked using permutation importance in a descending order. The weight reflects quantitative reduction in model performance with reshuffling of that column. PaO_2_: partial pressure of oxygen; FiO_2_: fraction of inspired oxygen; SaO_2_: oxygen saturation; P/F: PaO_2_ and FiO_2_ ratio; S/F: SaO_2_ and FiO_2_ ratio; Vent: mechanical ventilation.

**Figure 5:**
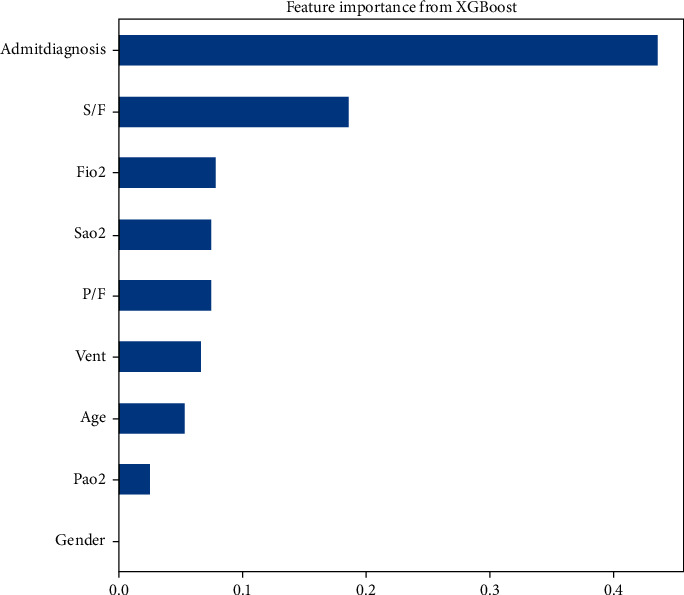
Feature importance ranking via the XGBoost built-in method. PaO_2_: partial pressure of oxygen; FiO_2_: fraction of inspired oxygen; SaO_2_: oxygen saturation; P/F: PaO_2_ and FiO_2_ ratio; S/F: SaO_2_ and FiO_2_ ratio; Vent: mechanical ventilation.

The SHAP plot demonstrates that the S/F ratio ranks as the strongest predictor for mortality amongst the physiologic variables of interest (Figure 3).  Figure 4 illustrates a similar trend as the S/F ratio remains the highest-ranking physiologic feature using the Eli5 library.  Figure 5 relays the feature importance using the XGBoost built-in feature importance method with the results remaining like SHAP and Eli5.

**Figure 6:**
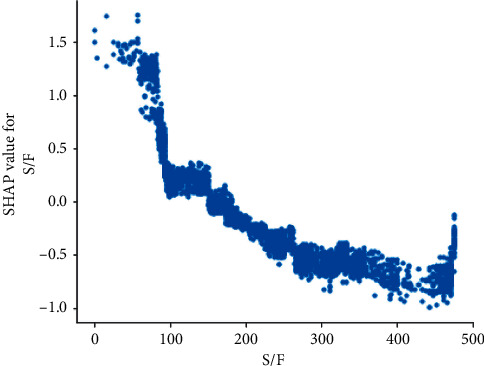
S/F partial dependence plot. The *y*-axis reflects the probability of death as a positive value denotes a higher probability. The *x*-axis demonstrates the S/F value. As S/F decreases, the probability of death increases. The probability of death appears to rise significantly below an S/F value of 150. S/F : SaO_2_/FiO_2_.

S/F ratio is the highest-ranking physiologic feature for predicting ICU mortality. This holds with the three different feature importance evaluation methods: SHAP, permutation importance, and the XGBoost feature importance. The S/F ratio partial dependence plot demonstrates a significant increase in mortality as the S/F drops below 200 (Figure 6).

## 4. Discussion

This study has described a supervised machine-learning model to predict ICU mortality using the standard parameters to assess hypoxia. The objective was to assess the feature importance from the classification model, but the importance is only valid if there is an accurate model. Model classifiers attained a strong AUC of 0.85, which reinforces confidence in the feature importance rankings. Feature importance rankings were created using three different methods, with the primary method being SHAP values. We employed an advanced machine-learning feature importance method in the form of SHAP. SHAP values are at the cutting edge for interpretable machine-learning models previously considered as black boxes by demonstrating feature importance in the context of every possible permutation combination. S/F ratio appeared to be the strongest physiologic predictor for ICU mortality based on all three modalities' feature importance rankings.

P/F ratio is the most used method for assessing hypoxic respiratory failure severity, especially ARDS. P/F calculation requires blood draws, increases costs, and can vary significantly even without oxygenation physiology changes [7]. Temporally, P/F has significant limitations as it is more labor-intensive and can cause theoretic delays in urgent interventions. Furthermore, since COVID-19 has changed the landscape of clinical care in the ICU, it can be argued that frequent ABG checks may potentially increase the infection risk for the ICU staff.

Continuous pulse oximetry is an accurate, continuous, noninvasive, and cost-effective method to assess hypoxia. It is a better indicator of oxygen delivery than P/F, as indicated by the oxygen delivery equation [23]. However, in our practice, it is not often used to make critical decisions for severe hypoxia, such as prone ventilation and neuromuscular blockade. S/F ratio provides all the benefits of SaO_2_ but provides a more nuanced understanding of the patient's hypoxia. Previous studies have shown a strong correlation between S/F and P/F, and S/F values can be accurately imputed from P/F and vice versa [11]. Hence, it would be reasonable to contemplate that a cheaper, safer, comparably accurate, and continuous disease monitoring method should be considered the primary means of disease evaluation.

**Figure 7:**
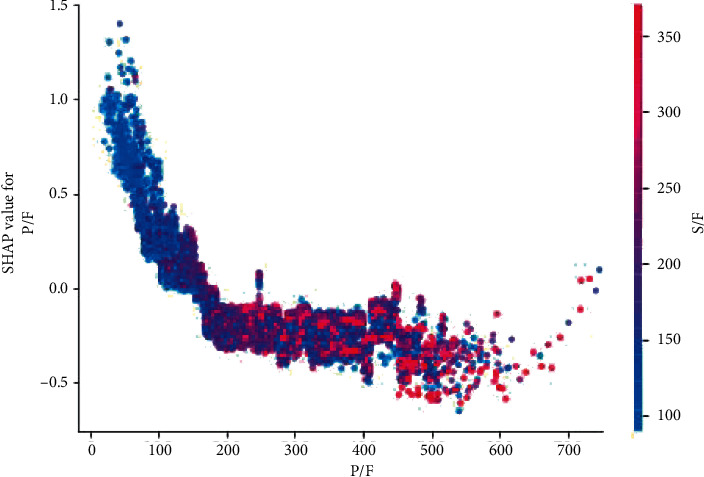
P/F partial dependence plot. The *y*-axis on the left reflects the probability of death as a positive value denotes a higher probability. The *y*-axis on the right shows the color scale of the value of S/F, which is superimposed upon the P/F plot. The *x*-axis demonstrates the S/F value. As P/F decreases, the probability of death increases. Probability of death appears to rise significantly below a P/F value of 200. S/F and P/F demonstrate a strong correlation, with both the quantitative value and changes in the probability of death. P/F: PaO_2_ and FiO_2_ ratio; S/F: SaO_2_ and FiO_2_ ratio.

A potential pitfall to consistent use of the S/F ratio to stratify hypoxic respiratory failure is the relative lack of knowledge of the proper cutoffs that guide interventions. The Kigali protocol provides cutoffs, and prior studies have shown linear and nonlinear relationships to P/F, which can be used [24]. We created a partial dependence plot to illustrate the cutoff at which mortality sharply increases, and this appears to be at an S/F ratio of about 200 (Figure 6). Additionally, in the partial dependence plots, we plot P/F against the probability of mortality with superimposed S/F values, which denotes the strong correlation between high and low values in the two measures (Figure 7). Table 1 illustrates the mean P/F and S/F ratios for patients grouped by mortality. Though our study aimed not to create specific cutoff values for S/F, this should be a future objective for a prospective evaluation.

The patient sample used in this study was multicentered and diagnostically heterogeneous. It included mechanically ventilated and nonmechanically ventilated patients, which distinguishes it from past studies evaluating the S/F ratio. To the best of our knowledge, there have been no prior studies that have used a machine-learning approach to compare the relative strengths of mortality prediction of S/F and P/F using modern feature importance methods.

The study has some limitations. The initial dataset had many missing values that were dropped, possibly introducing bias, though this coincided with assessing higher severity patients. The analysis is retrospective, hence warranting a prospective comparison of S/F and P/F. Also, feature importance evaluations should be interpreted with caution, but the present study's findings are only hypothesis-generating. Only one model was used to perform analysis, which could be strengthened by performing the same analysis on other models. Our study population is heterogeneous with the inclusion of all levels of oxygen and mechanical support, limiting our inferences' strength; for example, it is difficult to ascertain whether our conclusions would be valid for ECMO patients.

Additionally, pulse oximetry is not without limitations. Pulse oximetry is affected by many factors, including shock states, skin pigmentation, oximeter location, and anemia. Finally, we should avoid the common cognitive bias of false dichotomization and use S/F within the clinical scenario, which may require a blood gas for corroboration [25].

## 5. Conclusion

We hypothesized that using a noninvasive means for hypoxia evaluation through the S/F ratio would be noninferior to more invasive methods. This study demonstrates that, in the eICU database, S/F ratio appears to be a better predictor of ICU mortality than P/F. Combined with prior studies comparing S/F and P/F ratios, we believe that these findings could be potential practice-changers on a large scale.

## Figures and Tables

**Table 1:** Summary statistics of demographics and features.

	Overall	Alive	Expired	*P* value
*n*	33701	29363	4338	
Age, mean (SD)	63.2 (15.5)	62.8 (15.5)	65.9 (14.9)	<0.001
Vent, *n* (%) NM	5873 (17.4)	5477 (18.7)	396 (9.1)	<0.001
Vent, *n* (%) M	27828 (82.6)	23886 (81.3)	3942 (90.9)	
PaO_2_, mean (SD)	130.3 (85.2)	130.1 (83.7)	131.1 (94.5)	0.526
FiO_2_, mean (SD)	59.3 (26.3)	57.1 (25.5)	74.4 (26.3)	<0.001
SaO_2_, mean (SD)	95.4 (7.4)	96.0 (5.8)	91.6 (13.4)	<0.001
Gender, *n* (%) m	18991 (56.4)	16534 (56.3)	2457 (56.6)	0.695
Gender, *n* (%) f	14710 (43.6)	12829 (43.7)	1881 (43.4)	
APACHE, mean (SD)	74.4 (29.7)	69.6 (26.1)	107.0 (32.0)	<0.001
P/F, mean (SD)	239.3 (124.3)	246.1 (122.3)	193.8 (128.4)	<0.001
S/F, mean (SD)	197.3 (91.5)	204.1 (91.8)	150.6 (73.7)	<0.001

*n*: total number of individuals; SD: standard deviation; NM: nonmechanically ventilated patients; M: mechanically ventilated patients; PaO_2_: partial pressure of oxygen; FiO_2_: fraction of inspired oxygen; SaO_2_: oxygen saturation; m: male; f: female; APACHE: Acute Physiology and Chronic Health Evaluation score; P/F: PaO_2_ and FiO_2_ ratio; S/F: SaO_2_ and FiO_2_ ratio.

**Table 2:** Results from the cross-validation folds from the hyperparameter tuned training set.

	Accuracy	AUC	Recall	Prec.
0	0.8817	0.8507	0.1815	0.6395
1	0.8847	0.8493	0.1947	0.6782
2	0.8847	0.8459	0.2013	0.6703
3	0.8788	0.8325	0.1842	0.5957
4	0.8851	0.8334	0.2072	0.6774
5	0.8847	0.8472	0.2336	0.6455
6	0.8809	0.8398	0.2237	0.6018
7	0.8788	0.8445	0.1612	0.6125
8	0.8800	0.8305	0.1809	0.6180
9	0.8898	0.8532	0.2500	0.7037
Mean	0.8829	0.8427	0.2018	0.6443
SD	0.0033	0.0077	0.0259	0.0351

AUC: area under the curve; SD: standard deviation; Prec.: precision. Final scores are means of the ten folds.

## Data Availability

Data are available online in the eICU Collaborative Research Database.

## Supplementary Materials

Supplementary Materials Supplemental Figure 1: violin plot distribution of FiO_2_ plotted by mortality. The median FiO_2_ of survivors was approximately 50%, whereas expired patients had a median FiO_2_ of 80%. The thick gray bar in the middle of each plot represents the interquartile ranges above and below the median. Here we see that the 75% quartile of patient in the ALIVE group still had a lower FiO_2_ than the median in the expired group. FiO_2_: fraction of oxygen in inspired air. Supplemental Figure 2: violin plot distribution of SaO_2_ plotted by mortality. As expected, this violin plot shows that the patients who survived had higher oxygen saturations, with a greater number of patients with saturations closer to their median as compared to the EXPIRED group. SaO_2_: oxygen saturation. Supplemental Figure 3: violin plot distribution of S/F ratio plotted by mortality. The interquartile range extends over a wider range of S/F ratios in the patients who survived. The difference in the FiO_2_ ratios between the two groups in Figure 1 alone does not account for the median that is nearly double in the ALIVE versus the EXPIRED group. To further support this point, the number of patients with S/F ratios close to the median is greater than that in the FiO_2_ violin plot. S/F: ratio of oxygen saturation and fraction of oxygen in inspired air. Supplemental Figure 4: violin plot distribution of age plotted by mortality. Age was not a particularly important feature in predicting risk for ICU mortality in the patients included. Though the medians are different, there is a similar density of patients of the same age range in both groups. Supplemental Figure 5: violin plot distribution of P/F ratio plotted by mortality. Although P/F ratios span over a wider range of values than S/F ratios do based on the S/F's ratio's numerator being a percentage, P/F was less predictive of ICU mortality in a narrow range of values. P/F: ratio of partial pressure of oxygen and fraction of oxygen in inspired air. Supplemental Figure 6: distribution of admission diagnoses. Our inclusion criteria allowed for a wide range of ICU admission diagnoses in order to assess applicability in a variety of clinical situations. Though sepsis due to pulmonary etiologies and emphysema ranked high in a number of patients, cardiac etiologies were just as prevalent. Of course, any number of these patients could have had progression to ARDS. S-CABG = coronary artery bypass graft; SEPSISPULM = sepsis due to pulmonary etiology; CARDARREST = cardiac arrest; EMPHYSBRONC = emphysematous bronchitis; CHF = congestive heart failure; S = VALVAO = aortic valve replacement; RESPARREST = respiratory arrest; PNEUMBACT = bacterial pneumonia; M-RESOTHER = other diagnoses; SEPSISUTI = urinary tract infection due to sepsis; SEPSISGI = sepsis due to a gastrointestinal cause; CVASTROKE = cerebral vascular accident; SEPSISUNK = sepsis due to unknown cause; DKA  = diabetic ketoacidosis; AMI = acute myocardial infarction; SCABGAOV = coronary artery bypass graft and arctic valve replacement; IC = intraparenchymal hemorrhage; PNEUMASPIR = aspiration pneumonia; S-GIPERFR = gastrointestinal perforation. Supplemental Figure 7: proportion of missing values amongst parameters. The relative number of missing data points is illustrated in this bar chart. PaO_2_, FiO_2_, P/F ratios, and S/F ratios are all absent in equal numbers. The remaining patients' data points were utilized in our statistical analyses. S/F = SaO_2_/FiO_2_; P/F = PaO_2_/FiO_2_. Supplemental Figure 8: missing values bar chart prior to dropped PaO_2_ and FiO_2_. The percentage of missing value for each feature is shown in this bar graph. There were nearly equal numbers of missing data points for PaO_2_, FiO_2_, P/F ratio, and S/F ratio. Supplemental Figure 9: Precision, Recall, F1, and Accuracy for the hold-out test set from the hyperparameter optimized model. Performance was stratified by mortality with the mean as the final result. 0 = alive and 1 = expired. Supplemental Figure 10: partial dependence plot. This describes the relationship between a feature and its target. It includes a secondary feature that the primary feature most interacts with. Here the primary feature is the S/F ratio and the secondary feature is FiO_2_. The *x*-axis denotes the value of the primary feature, here the S/F ratio. The *y* value on the right is FiO_2_. An indirect sigmoid relationship is demonstrated here between the two. The fact that the values are dispersed close to one another to form this negative sigmoid curve denotes close feature interaction and just as the previous SHAP value bar graph, Figure 11, which demonstrated a large uptick in higher likelihood of mortality, particularly with S/F ratios less than 200, approximating the lower inflection point. FiO_2_ percentages greater than approximately 50% correspond to the lower inflection point as well. S/F = SaO_2_/FiO_2_. *s*
