# Supplementary material for: Mortality Prediction Using SaO2/FiO2 Ratio Based on eICU Database Analysis
Source: Crit Care Res Pract. 2021 Nov 8;2021:6672603. doi: 10.1155/2021/6672603 (PMC8592728; doi:10.1155/2021/6672603)
Supplement: Supplementary Materials — Supplemental Figure 1: violin plot distribution of FiO2 plotted by mortality. The median FiO2 of survivors was approximately 50%, whereas expired patients had a median FiO2 of 80%. The thick gray bar in the middle of each plot represents the interquartile ranges above and below the median. Here we see that the 75% quartile of patient in the ALIVE group still had a lower FiO2 than the median in the expired group. FiO2: fraction of oxygen in inspired air. Supplemental Figure 2: violin plot distribution of SaO2 plotted by mortality. As expected, this violin plot shows that the patients who survived had higher oxygen saturations, with a greater number of patients with saturations closer to their median as compared to the EXPIRED group. SaO2: oxygen saturation. Supplemental Figure 3: violin plot distribution of S/F ratio plotted by mortality. The interquartile range extends over a wider range of S/F ratios in the patients who survived. The difference in the FiO2 ratios between the two groups in Figure 1 alone does not account for the median that is nearly double in the ALIVE versus the EXPIRED group. To further support this point, the number of patients with S/F ratios close to the median is greater than that in the FiO2 violin plot. S/F: ratio of oxygen saturation and fraction of oxygen in inspired air. Supplemental Figure 4: violin plot distribution of age plotted by mortality. Age was not a particularly important feature in predicting risk for ICU mortality in the patients included. Though the medians are different, there is a similar density of patients of the same age range in both groups. Supplemental Figure 5: violin plot distribution of P/F ratio plotted by mortality. Although P/F ratios span over a wider range of values than S/F ratios do based on the S/F's ratio's numerator being a percentage, P/F was less predictive of ICU mortality in a narrow range of values. P/F: ratio of partial pressure of oxygen and fraction of oxygen in inspired air. Supplemental Figure [file 6672603.f1.docx]

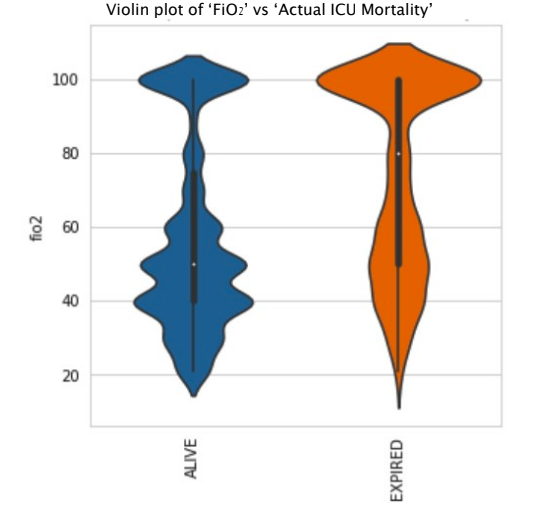


| **Supplemental Figure 1-** Violin plot distribution of Fi02 plotted by Mortality.  The median FiO2 of survivors was approximately 50% whereas expired patients had a median FiO2 of 80%. The thick gray bar in the middle of each plot represents the interquartile ranges above and below the median. Here we see that the 75% quartile of patients in the alive group still had a lower FiO2 than the median in the expired group. FiO2: fraction of oxygen in inspired air. |
| --- |


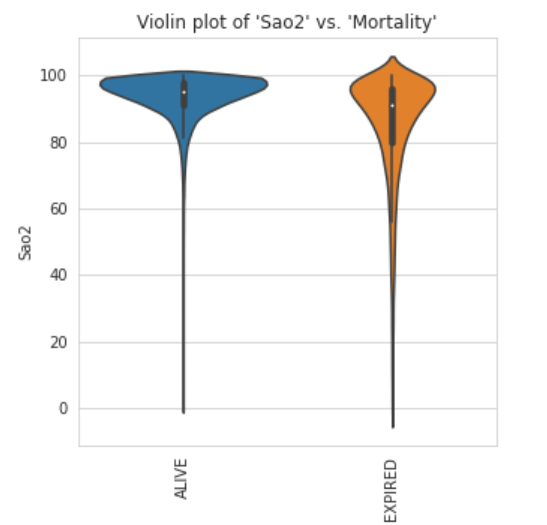


| **Supplemental Figure 2-**Violin plot distribution of Sao2 plotted by Mortality. As expected, this violin plot shows that the patients who survived had higher oxygen saturations with a greater number of patients with saturations closer to their median as compared to the EXPIRED group. SaO2: Oxygen saturation. |
| --- |


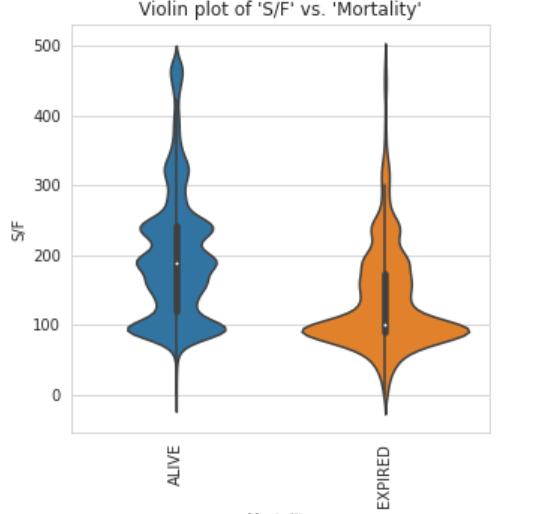


| **Supplemental Figure 3-** Violin plot distribution of S/F ratio plotted by Mortality. The interquartile range extends over a wider range of S/F ratios in the patients who survived. The difference in the FiO2 ratios between the two groups in Figure 1 alone does not account for the median that is nearly double in the ALIVE vs the EXPIRED group. To further support this point, the number of patients with S/F ratios close to the median is greater than in the FiO2 violin plot. S/F: ratio of oxygen saturation and fraction of oxygen in inspired air. |
| --- |


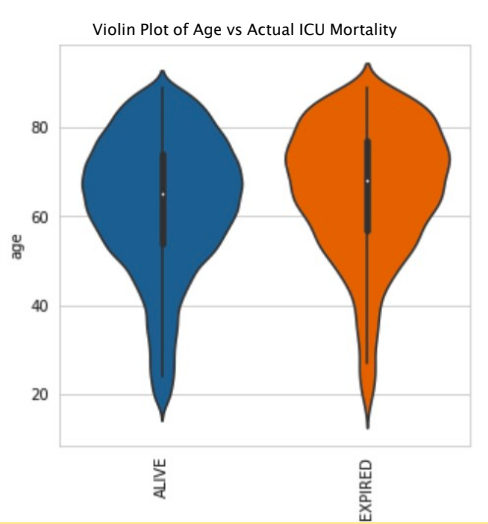


| **Supplemental Figure 4-** Violin plot distribution of age plotted by Mortality. Age was not a particularly important feature in predicting risk for ICU mortality in the patients included. Though the medians are different, there is a similar density of patients of the same age range in both groups. |
| --- |


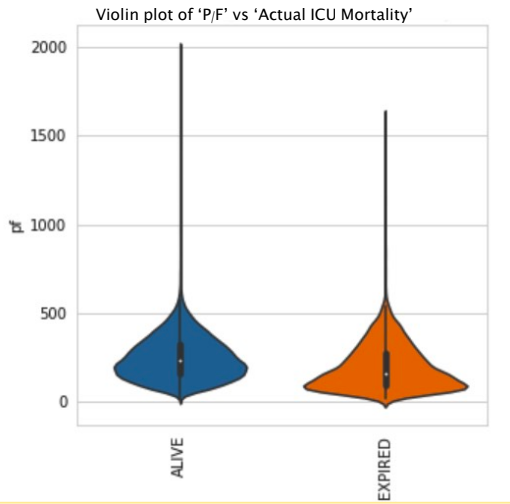


| Supplemental Figure 5- Violin plot distribution of P/F ratio plotted by Mortality. Although P/F ratios span over a wider range of values than S/F ratios do based on the S/F's ratio's numerator being a percentage, P/F was less predictive of ICU mortality in a narrow range of values. P/F : ratio of partial pressure of oxygen and fraction of oxygen in inspired air. |
| --- |


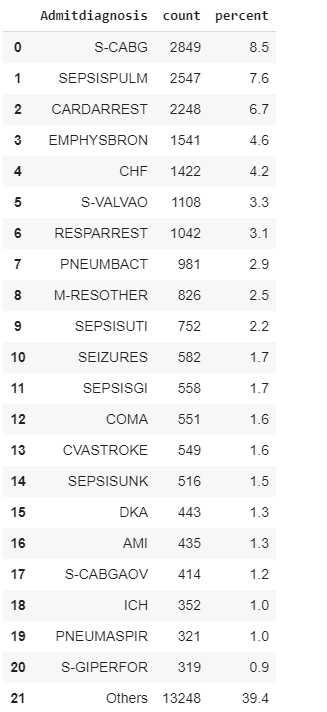


| **Supplemental Figure 6-** Distribution of Admission Diagnoses. Our inclusion criteria allowed for a wide range of ICU admission diagnoses in order to assess applicability in a variety of clinical situations. Though sepsis due to pulmonary etiologies and emphysema ranked high in number of patients, cardiac etiologies were just as prevalent. Of course, any number of these patients could have had progression to ARDS. S-CABG= Coronary artery bypass graft. SEPSISPULM= Sepsis due to pulmonary etiology. CARDARREST= Cardiac arrest. EMPHYSBRONC= Emphysematous bronchitis. CHF= Congestive Heart Failure. S=VALVAO= Aortic valve replacement. RESPARREST = Respiratory arrest. PNEUMBACT= Bacterial Pneumonia. M-RESOTHER= Other diagnoses. SEPSISUTI = Urinary tract infection due to sepsis. SEPSISGI = Sepsis due to a gastrointestinal cause. CVASTROKE= Cerebral vascular accident. SEPSISUNK = Sepsis due to unknown cause. DKA= Diabetic Ketoacidosis. AMI = Acute myocardial infarction. SCABGAOV = Coronary artery bypass grat and arctic valve replacement. IC = Intraparenchymal hemorrhage. PNEUMASPIR= Aspiration pneumonia. S-GIPERFR= Gastrointestinal perforation. |
| --- |


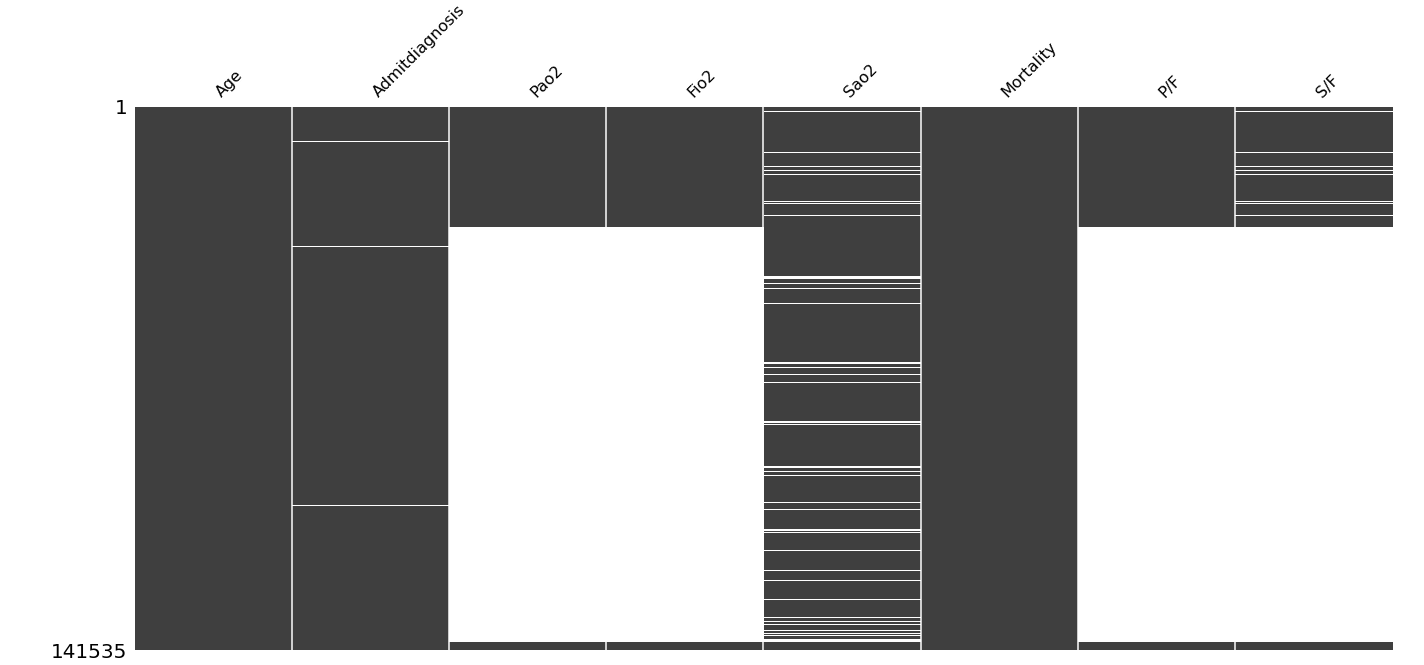


| **Supplemental Figure 7-** Proportion of missing values amongst parameters. The relative number of missing data points is illustrated in this bar chart. PaO2, FiO2, P/F ratios, and S/F ratios are all absent in equal numbers. The remaining patients' data points were utilized in our statistical analyses.  S/F= Sao2/Fio2. P/F= PaO2/FiO2 |
| --- |


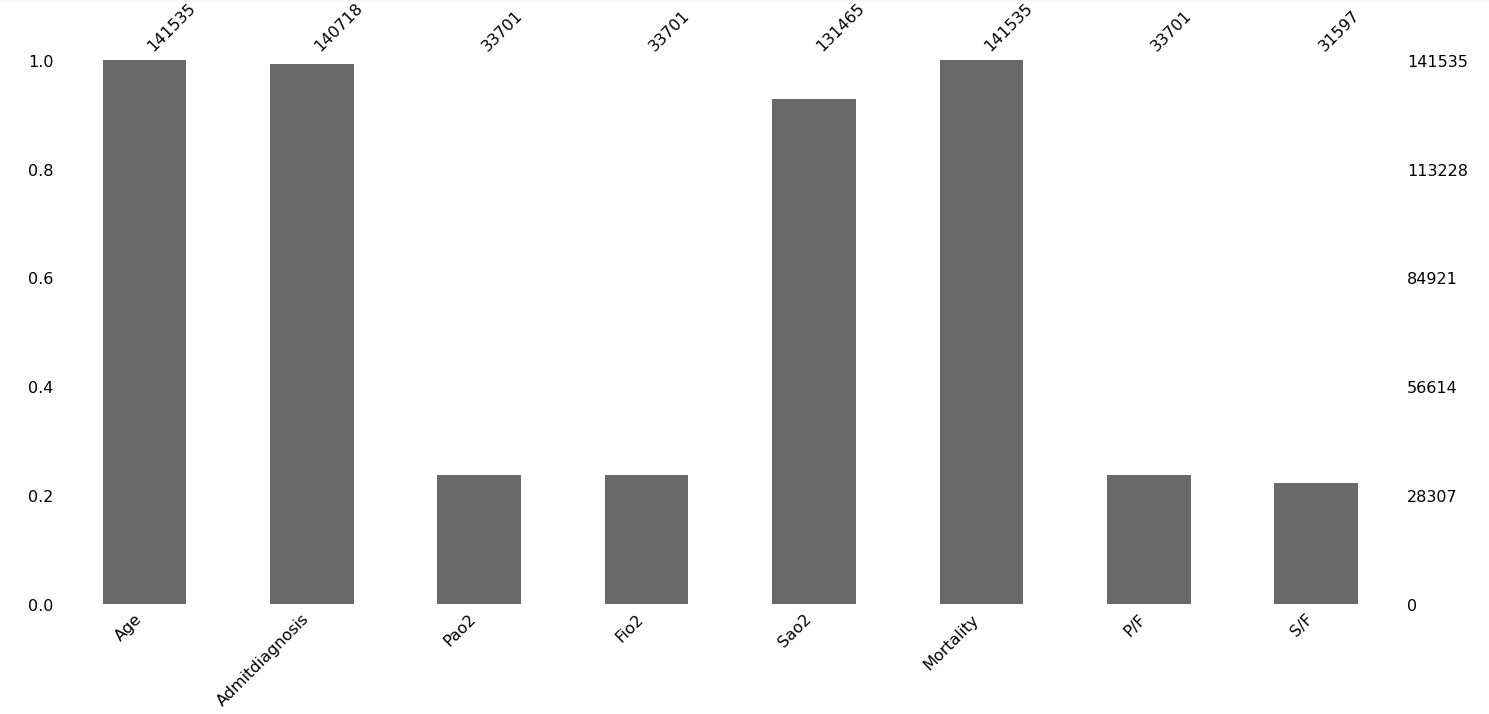


| **Supplemental Figure 8-** Missing values bar chart prior to dropped pao2 and fio2. The percentage of missing value for each feature is shown in this bar graph. There were nearly equal numbers of missing data points for PaO2, FiO2, P/F ratio, and S/F ratio. |
| --- |


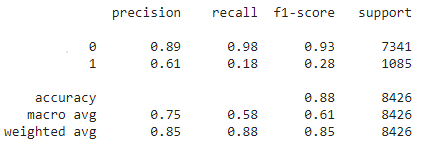


| **Supplemental Figure 9-** Precision, Recall, F1 and Accuracy for the hold-out test set from the hyperparameter optimized model.  Performance stratified by Mortality with the mean as the final result.  0= Alive, 1=Expired |
| --- |


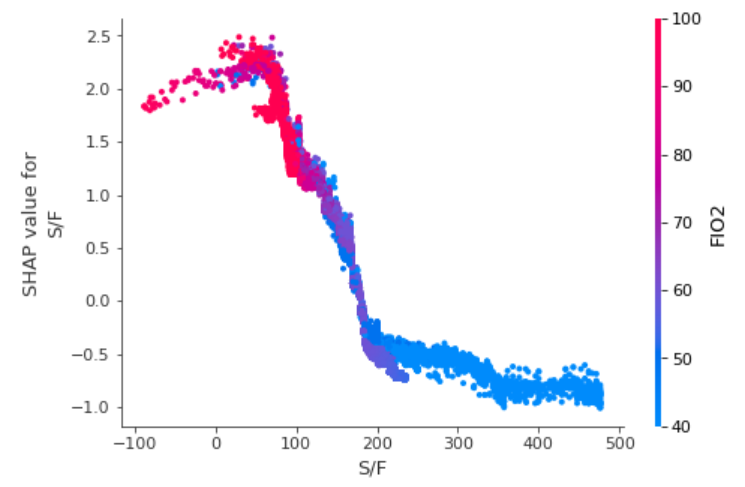


| **Supplemental Figure 10-** Partial Dependence Plot. This describes the relationship between a feature and its target. It includes a secondary feature that the primary feature most interacts with. Here the primary feature is the S/F ratio and the secondary feature is FiO2. The x-axis denotes the value of the primary feature, here, the S/F ratio. The y value on the right is FiO2. An indirect sigmoid relationship is demonstrated here between the two. The fact that the values are dispersed close to one another to form this negative sigmoid curve denotes close feature interaction. And just as the previous SHAP value bar graph, Figure 11, which demonstrated a large uptick in higher likelihood of Mortality, particularly with S/F ratios less than 200, approximating the lower inflection point. FiO2 percentages greater than approximately 50% correspond to the lower inflection point as well. S/F= Sao2/Fio2 |
| --- |
